# Supplementary material for: Novel Driver Strength Index highlights important cancer genes in TCGA PanCanAtlas patients
Source: PeerJ. 2022 Aug 11;10:e13860. doi: 10.7717/peerj.13860 (PMC9375969; doi:10.7717/peerj.13860)
Supplement: Supplemental Information 2 [file peerj-10-13860-s002.zip › COHORTS/genes plots/cohorts/2021_8_16_14_9_distribution_cohorts_STAD.pdf]

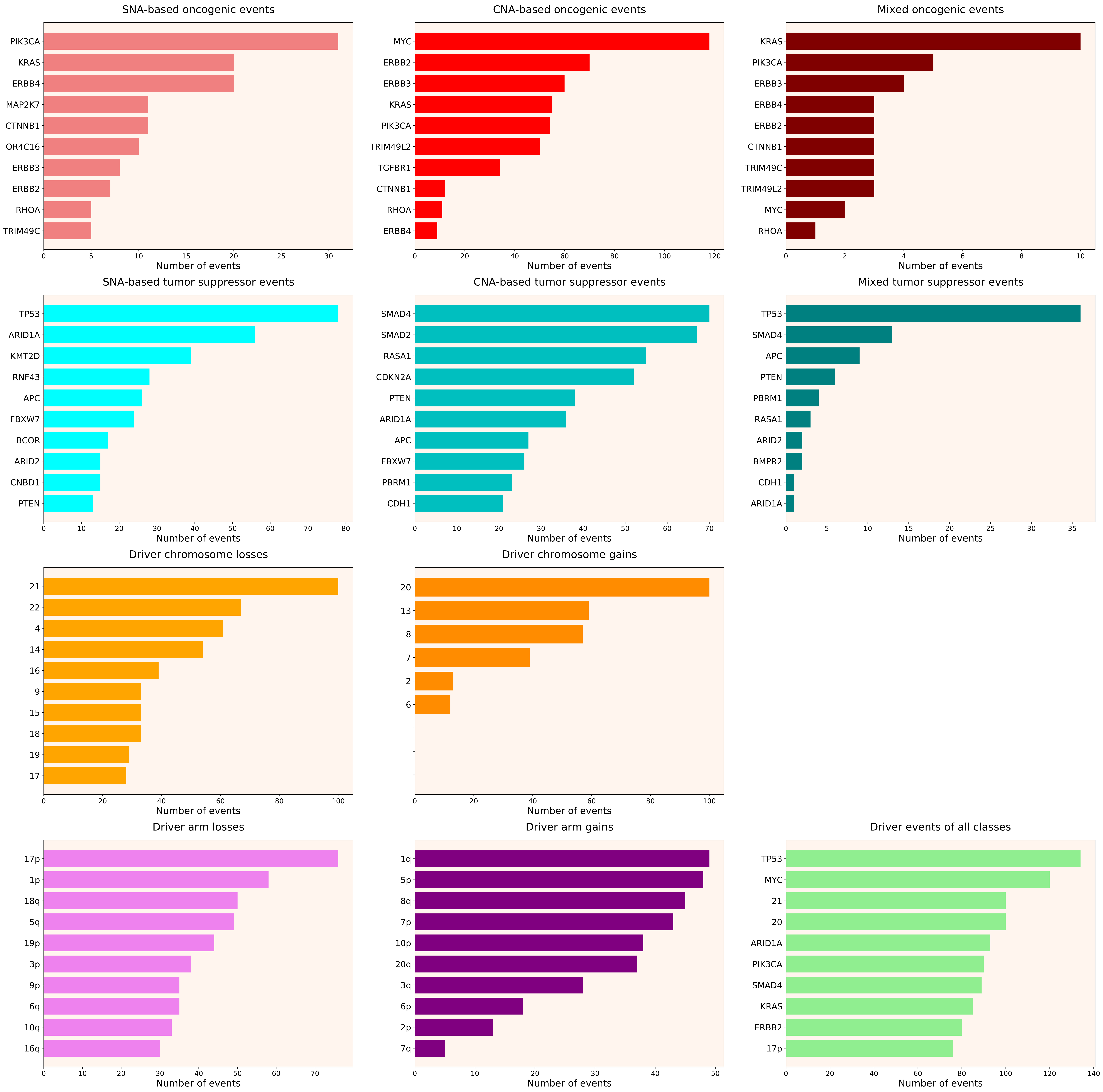
SNA-based oncogenic events
PIK3CA
KRAS
ERBB4
MAP2K7
CTNNB1
OR4C16
ERBB3
ERBB2
RHOA
TRIM49C
0 5 10 15 20 25 30
Number of events
CNA-based oncogenic events
MYC
ERBB2
ERBB3
KRAS
PIK3CA
TRIM49L2
TGFBR1
CTNNB1
RHOA
ERBB4
0 20 40 60 80 100 120
Number of events
Mixed oncogenic events
KRAS
PIK3CA
ERBB3
ERBB4
ERBB2
CTNNB1
TRIM49C
TRIM49L2
MYC
RHOA
0 2 4 6 8 10
Number of events
SNA-based tumor suppressor events
TP53
ARID1A
KMT2D
RNF43
APC
FBXW7
BCOR
ARID2
CNBD1
PTEN
0 10 20 30 40 50 60 70 80
Number of events
CNA-based tumor suppressor events
SMAD4
SMAD2
RASA1
CDKN2A
PTEN
ARID1A
APC
FBXW7
PBRM1
CDH1
0 10 20 30 40 50 60 70
Number of events
Mixed tumor suppressor events
TP53
SMAD4
APC
PTEN
PBRM1
RASA1
ARID2
BMPR2
CDH1
ARID1A
0 5 10 15 20 25 30 35
Number of events
Driver chromosome losses
21
22
4
14
16
9
15
18
19
17
0 20 40 60 80 100
Number of events
Driver chromosome gains
20
13
8
7
2
6
0 20 40 60 80 100
Number of events
Driver arm losses
17p
1p
18q
5q
19p
3p
9p
6q
10q
16q
0 10 20 30 40 50 60 70
Number of events
Driver arm gains
1q
5p
8q
7p
10p
20q
3q
6p
2p
7q
0 10 20 30 40 50
Number of events
Driver events of all classes
TP53
MYC
21
20
ARID1A
PIK3CA
SMAD4
KRAS
ERBB2
17p
0 20 40 60 80 100 120 140
Number of events
